# Stress Hyperglycemia Ratio Predicts 28-Day Mortality in Patients with AMI Cardiogenic Shock: Insights from a Large-Scale Cohort

**DOI:** 10.3390/jcm15114271

**Published:** 2026-06-01

**Authors:** Jiandu Yang, Jun Zhang, Shuhan Zhou, Xinlin Luo, Chao Guo, Kai Liu

**Affiliations:** 1 State Key Laboratory of Cardiovascular Disease, Fuwai Hospital, National Center for Cardiovascular Diseases, Chinese Academy of Medical Sciences and Peking Union Medical College, Beijing 100037, China; jianduyang@yahoo.com (J.Y.); zhangjun-cv@263.net (J.Z.);; 2 Fuwai Hospital, Chinese Academy of Medical Sciences, Shenzhen 518001, China

**Keywords:** acute myocardial infarction, cardiogenic shock, stress hyperglycemia ratio, blood glucose fluctuation, risk scoring system

## Abstract

**Background:** The prognostic impact of the stress hyperglycemia ratio (SHR24) and blood glucose (BG) fluctuation within 24 h (ΔBG_24_) on cardiogenic shock complicating acute myocardial infarction (AMICS) remains unclear. This study evaluated the prognostic value of the SHR_24_ and ΔBG_24_ for short-term mortality in AMICS. **Methods:** We retrospectively analyzed AMICS patients from 2016 to 2022. The primary outcome was 28-day all-cause mortality. Associations were evaluated using Kaplan–Meier (K-M) curves, Cox proportional hazard regression, and restricted cubic spline (RCS) analyses. **Results:** A total of 179 participants with AMICS were enrolled. The mean age was 64.44 ± 12.61 years. K-M curves showed significant differences in survival across ΔBG_24_ and the SHR_24_ (*p* < 0.001). Cox regression identified culprit vessel final blood flow (HR = 0.58, 95% CI: 0.43–0.80), the SHR_24_ (HR = 2.99, 95% CI: 1.61–5.57), and CardShock score (HR = 1.52, 95% CI: 1.18–1.96) as independent predictors of mortality. RCS analysis confirmed a linear correlation between the SHR_24_ and all-cause mortality (*p* = 0.001). Adding the SHR_24_ and ΔBG_24_ to the IABP-SHOCK II score increased the AUC by 0.093 (13.29%, *p* = 0.013) and 0.080 (11.43%, *p* = 0.016), respectively. For the CardShock score, they increased the AUC by 0.091 (12.07%, *p* = 0.002) and 0.056 (7.43%, *p* = 0.03). Decision curve analysis further confirmed that both the SHR_24_ and ΔBG_24_ improved clinical decision-making benefit. **Conclusions:** The SHR_24_ and ΔBG_24_ are reliable predictors of short-term prognosis in patients with AMICS.

## 1. Background

Cardiogenic shock (CS), characterized by low cardiac output resulting in life-threatening organ hypoperfusion and tissue hypoxia, is a complex and critically life-threatening clinical condition. It persists as the primary factor accounting for in-hospital deaths in individuals with acute myocardial infarction (AMI) [1,2,3]. Therefore, early identification of high-risk patients and the discovery of more risk factors are essential for improving patient prognosis [4,5].

CS often leads to dysregulation of hormone distribution and, consequently, contributes to stress-induced hyperglycemia. Recently, numerous studies have shown that stress hyperglycemia is associated with increased morbidity and mortality in patients with myocardial infarction [6,7], heart failure [8,9], chronic obstructive pulmonary disease, cerebrovascular disease and critical illness [10,11]. In this study, the maximum change in BG within 24 h (ΔBG_24_, the highest blood glucose minus the lowest blood glucose within 24 h) and the SHR (expressed as SHR_24_), calculated based on the highest blood glucose value within 24 h, were included as the research objects of stress-induced blood glucose. As an indicator of acute hyperglycemic status, the SHR has been confirmed to be associated with a poor short-term outcome in both diabetic and non-diabetic patients suffering from heart failure [12], AMI [13], and stroke [14]. However, it remains uncertain whether ΔBG_24_ and the SHR_24_ can effectively predict outcomes in patients with cardiogenic shock complicating acute myocardial infarction (AMICS). This study provides new evidence of the predictive value of ΔBG24 and the SHR24 in this high-risk population. Among current scoring systems, CardShock [15] and IABP-SHOCK II risk scores [16] have been rigorously validated through recent studies, establishing their credibility in predicting short-term prognosis [17,18,19]. Among these scores, the glucose level on admission has been incorporated into some risk scoring systems, such as IABP-SHOCK II. However, while glucose at admission is a traditional predictor, it can be influenced by several variables, including pharmacological interventions and chronic glucose levels. This variability may limit its ability to provide a complete assessment of hyperglycemia, particularly concerning glucose elevations that arise from acute critical conditions. Therefore, there is a need to consider additional factors such as ΔBG_24_ and the SHR_24_ to better capture the nuances of hyperglycemia in such clinical contexts.

## 2. Method

### 2.1. Study Population

This study included patients with AMICS admitted to our hospital from January 2016 to December 2022. The diagnosis of cardiogenic shock was confirmed within one hour after admission. To evaluate the impact of the SHR_24_ on short-term prognosis after shock diagnosis, a retrospective analysis of these patients’ clinical data was conducted.

The study was conducted in accordance with the ethical standards of the institutional and national research committees and the 1964 Helsinki Declaration and its later amendments, or comparable ethical standards. It was approved by the Ethics Committee of Fuwai Hospital, Chinese National Center of Cardiovascular Disease. All patients provided written informed consent.

### 2.2. Definition

A confirmed diagnosis of myocardial infarction was made at the discretion of the treating physician based on the universal definition of AMI [20]. There is an acute myocardial injury with clinical evidence of acute myocardial ischemia. This is with the detection of a rise and/or fall of cardiac troponin I values with at least one value above the 99th percentile Upper Reference Limit (URL) and at least one of the following: (1) symptoms of myocardial ischemia; (2) new ischemic electrocardiogram (ECG) changes; (3) development of pathological Q waves; (4) imaging evidence of new loss of viable myocardium or new regional wall motion abnormality in a pattern consistent with an ischemic etiology; (5) identification of a coronary thrombus by angiography or autopsy.

Cardiogenic shock diagnostic criteria are as follows: systolic blood pressure (SBP) measurements of <90 mm Hg for ≥30 min or use of pharmacological and/or mechanical support to maintain an SBP ≥ 90 mm Hg. Evidence of end-organ hypoperfusion varied across trials but typically included urine output < 30 mL/h, cool extremities, altered mental status, and/or serum lactate > 2.0 mmol/L [4]. Moreover, uniform diagnostic criteria were consistently applied to all enrolled patients at baseline.

The SHR_24_ was calculated by the following formula: the highest glucose value within the first 24 h plasma glucose (mmol/L)/1.59 × Glycated Hemoglobin (HbA1c) (%) − 2.59 [21]. The glucose value and HbA1c were measured by Fuwai Hospital. All patients were classified into three groups by tertiles of ΔBG_24_ and the SHR_24_. Baseline glucose values were determined from blood samples collected within the first hour of hospital admission. However, the 24 h blood glucose variation, namely ΔBG_24_ and the SHR_24_, was defined as the maximum glucose fluctuation observed within 24 h after admission.

### 2.3. Data Collection

The demographic profiles and medical backgrounds of the patients, including hypertension, dyslipidemia, chronic renal disease status, stroke history, and coronary revascularization procedures, were systematically extracted from their medical records. This was achieved through standardized definitions by two experienced data inspectors. A comprehensive array of clinical data was also collected, encompassing diastolic blood pressure (DBP) and systolic blood pressure (SBP) at the time of admission, respiratory rate (RR), heart rate (HR), Killip classification, instances of hemodynamic alterations, occurrence of malignant arrhythmias, mechanical complications, utilization of intra-aortic balloon pump (IABP), and necessity for mechanical ventilation.

Moreover, blood test results were meticulously collected, covering routine hematological parameters (such as platelet count, white blood cell count, and hemoglobin levels), biochemical indicators (including albumin, transaminases, bilirubin, creatinine, electrolytes, and lipid profiles), coagulation function assessments, and blood gas analyses (encompassing pH, partial pressures of carbon dioxide and oxygen, lactate levels, and bicarbonate concentrations). Cardiac markers were also included in this extensive data collection. Notably, all these indicators were recorded based on their worst values observed within the crucial first 24 h following the diagnosis of AMICS.

Additionally, to further evaluate each patient’s condition, the IABP-SHOCK II score and CardShock score were calculated, providing valuable insights into their prognosis and potential treatment requirements.

Additionally, through case record documentation or telephone follow-up with patients or their families, the all-cause mortality of patients within 28 days of follow-up data was investigated.

### 2.4. Statistical Analysis

For continuous variables, the Shapiro–Wilk (W) test was initially employed to ascertain their normal distribution. Variables adhering to normal distribution were subsequently presented as mean ± standard deviation (SD), with *p*-values derived from one-way ANOVA. Conversely, non-normally distributed continuous variables were reported as median (Q1, Q3), and comparisons among groups were made using the Mann–Whitney U test. Categorical variables were displayed as frequency counts and percentages, with comparisons conducted through Pearson’s chi-square test.

For survival analysis, the Kaplan–Meier survival curve was utilized to visualize survival rates, and differences in survival between groups were evaluated using the log-rank test. ROC curves for combined indicators were plotted, facilitating comparisons among them. To verify the value of the SHR_24_ and ΔBG_24_ in clinical decision-making, a decision curve analysis (DCA) was also performed.

Cox regression analysis was performed to identify the risk factors for 28-day mortality in patients. In this multivariable Cox regression model, candidate variables were selected based on previously published evidence and clinical biological plausibility. Variables previously confirmed to be associated with long-term survival and prognosis in patients with acute myocardial infarction complicated by cardiogenic shock were included directly in the regression model, without relying solely on univariable statistical significance. The overall significance of the multivariable Cox model was evaluated using the omnibus likelihood ratio test.

To delve deeper into the relationship between ΔBG_24_, the SHR_24_, and mortality, Cox proportional hazards regression models with restricted cubic spline (RCS) analyses and 4 knots were employed. In these models, we adjusted for potential confounding factors such as age, sex, hypertension, mitral regurgitation, ventricular tachycardia (VT)/ventricular fibrillation (VF), invasive mechanical ventilation, blood pressure, shock index, hematocrit (HCT), serum creatinine (Scr), albumin (Alb), and Alanine Aminotransferase (ALT). When the relationship was nonlinear, we estimated the threshold and identified the inflection point with the highest likelihood.

Statistical significance was set at a *p*-value of <0.05. All statistical analyses were carried out using GraphPad Prism 9.5.1 (GraphPad Software, Boston, MA, USA) software, SPSS software version 22.0 (SPSS Inc., Chicago, IL, USA), and R software (version 4.3.2).

## 3. Results

### 3.1. Baseline Characteristics

A total of 179 patients with AMICS who were admitted to our hospital from January 2016 to December 2022 were enrolled in this study. The baseline characteristics of these patients are presented in Table 1. The mean age of this cohort was 64.44 ± 12.61 years, with 124 males and 55 females. The 28-day mortality rate was 60.34%. The exclusion criteria were as follows: (1) age less than 18 years; (2) hypotension caused by malignant arrhythmia; (3) low cardiac output or shock occurring after cardiac or non-cardiac surgery; (4) incomplete case or follow-up data.

Notably, compared with survivors, non-survivors exhibited a significantly older age distribution, along with a higher prevalence of hypertension, elevated creatinine, elevated liver enzyme levels, and higher IABP-SHOCK II and CardShock scores. Additionally, this group showed a higher incidence of VT/VF and mitral regurgitation and a greater need for mechanical ventilation, coupled with compromised hemodynamics, lower arterial blood pH, and decreased albumin levels.

A total of 129 patients underwent emergency coronary angiography, including 71 non-survivors and 58 survivors. Analysis of coronary angiography characteristics revealed that the non-survivors had a disproportionately higher involvement of the circumflex artery as the culprit’s vessel. Conversely, the survivors displayed a higher rate of stent implantation. Furthermore, the non-survivors exhibited a strikingly higher proportion of final thrombolysis in myocardial infarction (TIMI) flow below grade 2 (70.40% vs. 5.20%, *p* < 0.001). Nevertheless, the two groups were comparable with respect to the prevalence of triple-vessel disease and the distribution of culprit vessels, including the left main, left anterior descending (LAD), and right coronary artery (RCA), as shown in Table 2. In terms of blood glucose fluctuation indicators, the ΔBG_24_ and SHR_24_ in the non-survivors were significantly higher than those in survivors.

### 3.2. Relationship Between Stress-Induced Glucose Changes and Mortality

**Figure 1:**
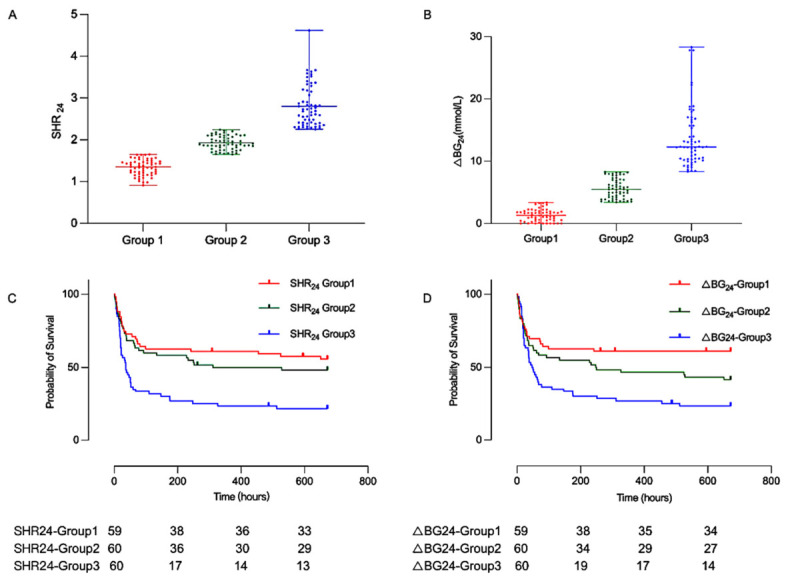
(**A**) The distribution of the SHR_24_ in three groups; (**B**) the distribution of ΔBG_24_ in three groups; (**C**) Kaplan–Meier survival curve of AMICS with the SHR_24_ within 28 days (672 h); (**D**) Kaplan–Meier survival curve of AMICS with ΔBG_24_ within 28 days (672 h).

Patients were categorized into three groups according to their average levels of ΔBG_24_ and the SHR_24_, with their baseline characteristics illustrated in Figure 1A,B, respectively. After the definitive diagnosis of AMICS, significant differences were observed in the Kapla–-Meier (K-M) survival curves among the three groups categorized by ΔBG_24_ (*p* < 0.001) and the SHR_24_ (*p* < 0.001).

To further analyze the impact of ΔBG_24_ and the SHR_24_ on the prognosis of patients with AMICS, this study included indicators such as hypertension, T2DM, ventricular arrhythmias (including ventricular tachycardia and ventricular fibrillation), shock index, VIS, emergency PCI, IABP support, final TIMI grade of blood flow, SHR_24_, and CardShock score into the regression equation. Cox regression analysis revealed that only the final blood flow of the culprit vessel (HR = 0.584, 95% CI: 0.426–0.801), SHR_24_ (HR = 2.991, 95% CI: 1.607–5.567), and CardShock score (HR = 1.517, 95% CI: 1.176–1.958) were independent predictors with statistical significance. Among them, a higher final blood flow in the culprit’s vessel served as a protective factor, while higher SHR_24_ and CardShock scores were both risk factors (Table 3).

### 3.3. Linear Relationships Between SHR_24_ and Short-Term Mortality

Cox proportional hazards regression models with RCS were used to evaluate the linear correlation between the SHR_24_ and short-term mortality in patients with AMICS.

**Figure 2:**
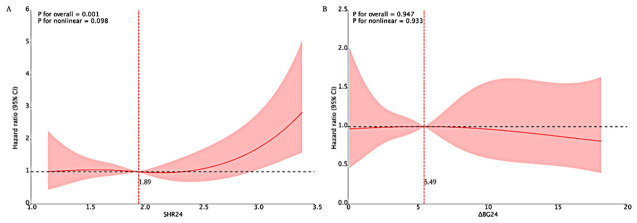
Restricted cubic spline curve for the hazard ratio of the SHR_24_ and ΔBG_24_. SHR_24_ 1.89 and ΔBG_24_ 5.49, represented by the vertical dotted lines, had an estimated hazard ratio of 1.0. The horizontal dotted line represents the hazard ratio of 1.0. (**A**) Restricted cubic spline of the SHR_24_ for all-cause mortality. (**B**) Restricted cubic spline of ΔBG_24_ for all-cause mortality.

The RCS analysis revealed a statistically significant linear correlation between the SHR_24_ and mortality (*p* = 0.001), while nonlinearity was not supported (*p* = 0.10), as depicted in Figure 2A. In the RCS analysis using the median value of the SHR_24_ (1.89) as the reference, the hazard ratio (HR) remained close to 1, with no significant difference when the SHR_24_ was below 1.89. However, the risk of adverse outcomes increased sharply once the SHR_24_ exceeded 1.89, suggesting a threshold effect. This association remained significant even after accounting for various confounding variables such as age, sex, hypertension status, mitral regurgitation, VT/VF, the need for invasive mechanical ventilation, blood pressure, shock index, and HCT, SCR, Alb, and ALT levels.

Similarly, the median value of ΔBG_24_ (5.49) was also selected as the reference point (HR = 1) in the RCS analysis. However, no significant overall or nonlinear association was observed between ΔBG_24_ and adverse outcomes. The HR curve remained stable around 1 across the entire range of ΔBG_24_, with overall and nonlinearity tests showing the *p*-values (*p* for overall = 0.95, *p* for nonlinear = 0.93, respectively), as illustrated in Figure 2B.

### 3.4. Improvement in Current Scoring Systems

**Figure 3:**
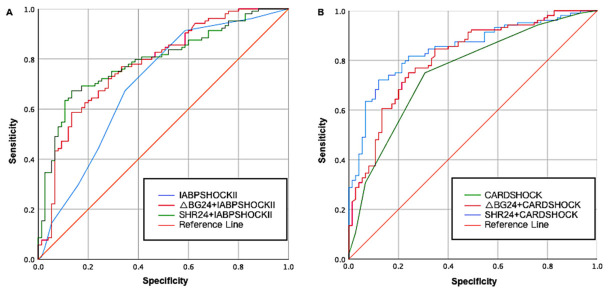
(**A**) ROC curve for IABP-SHOCK II scoring, ΔBG_24_ + IABP-SHOCK II, SHR_24_ + IABP-SHOCK II; (**B**) ROC curve for CardShock scoring, ΔBG_24_ + CardShock, SHR_24_ + CardShock II.

Based on the IABP-SHOCK II, CardShock scores, and the 28-day prognosis of patients, ROC curves were plotted separately, with areas under the ROC curves (AUCs) of 0.70 (95% CI: 0.63–0.77) and 0.75 (95% CI: 0.69–0.82), respectively. The ROC curves were used to calculate the cut-off values, sensitivity, and specificity, as shown in Figure 3 and Table 4. When ΔBG_24_ and the SHR_24_ were used in combination with the two scoring systems for diagnostic purposes and ROC curves were plotted for comparison with the original scores, it was found that ΔBG_24_ and the SHR_24_ could further improve the discrimination of the IABP-SHOCK II and CardShock scores; the SHR_24_ and ΔBG_24_ can increase the area under the ROC curve of the IABP-SHOCK II scoring system by 0.093 (13.29%, *p* = 0.013) and 0.080 (11.43%, *p* = 0.016), respectively, and can also increase the area under the ROC curve of the CardShock scoring system by 0.091 (12.07%, *p* = 0.002) and 0.056 (7.43%, *p* = 0.03), respectively.

**Figure 4:**
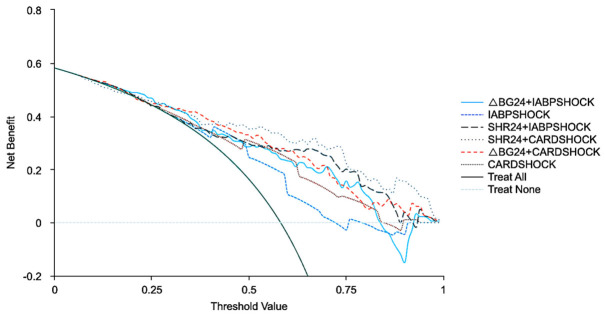
Decision curve analysis of the IABP-SHOCK II and CardShock scores with and without SHR_24_/ΔBG_24_.

To further evaluate the clinical utility and decision-making value of the SHR_24_/ΔBG_24_, decision curve analysis (DCA) was conducted to assess the net clinical benefit across different threshold probabilities. The results demonstrated that the model incorporating the SHR_24_/ΔBG_24_ yielded a higher net clinical benefit across a wide range of threshold probabilities compared with the original IABP-SHOCK II and CardShock scores. The new model demonstrated greater clinical decision-making value, further supporting the incremental prognostic value of the SHR_24_ and ΔBG_24_ (Figure 4).

## 4. Discussion

This study is among the few that examine the relationship between the SHR_24_ and the prognosis of the AMICS population, thereby enhancing the prognostic assessment provided by current shock risk scores. Our study suggests that even after adjusting for potential confounding variables, the SHR_24_ was independently associated with all-cause and cardiovascular mortality in AMICS patients, exhibiting a linear relationship for all-cause mortality.

In fact, the SHR, first described in 2015 [21], provides a measure of an individual’s acute hyperglycemia relative to their HbA1c and has been extensively studied across different diseases, highlighting its potential as a valuable prognostic indicator for cardiovascular events [22,23,24,25]. To be more specific, in patients with AMI, elevated SHR levels were associated with short-term mortality, independent of their baseline diabetes status. As is proven in the MIMIC-IV database, an elevated SHR is significantly associated with 1-year and long-term all-cause mortality, especially in those without diabetes, and the results are consistent in both American and Chinese cohorts [26,27]. In critically ill patients with sepsis, a higher SHR was associated with increased 28-day all-cause mortality and incidence of new-onset atrial fibrillation. This indicates that the SHR could be a useful prognostic biomarker for predicting outcomes and guiding therapeutic interventions [28,29]. This further supports the SHR as a significant prognostic biomarker in acute cardiovascular events.

In this study, it was found that the SHR_24_ is an important predictor of clinical prognosis. Using survival curves, substantial differences in clinical prognosis were observed among groups with different SHR_24_ and ΔBG_24_ levels, with higher SHR_24_ levels correlating with poorer prognosis. However, upon conducting Cox regression analysis, it was found that the SHR_24_ was more clinically useful than ΔBG_24_, and a linear correlation was observed between the SHR_24_ and short-term mortality. In contrast, no significant correlation was observed between ΔBG_24_ and short-term mortality. Furthermore, the SHR_24_ was more valuable than ΔBG_24_ in improving clinical scoring systems. In conclusion, this study found that the SHR_24_ is more predictive of patient prognosis than ΔBG_24_ in patients with AMICS.

Concurrent activation of inflammatory pathways upregulates gluconeogenesis and glycogenolysis, resulting in elevated blood glucose levels, as seen in myocardial infarction or CS complicating AMI. The exact reasons why the SHR_24_ is associated with a poorer prognosis in AMICS remain unclear. However, possible explanations include the following: high glucose levels increase reactive oxygen species (ROS) production, leading to oxidative stress that damages cellular components, including lipids, proteins, and DNA. This impairment of endothelial function can worsen outcomes in cardiovascular diseases [28]. Additionally, hyperglycemia weakens the immune system, reducing the effectiveness of neutrophils and macrophages, which increases infection susceptibility, particularly in critically ill patients or those undergoing surgery [30].

Furthermore, elevated blood glucose levels are linked to significant metabolic imbalances, including disruptions in insulin signaling and glucose metabolism. These imbalances can result in energy deficits in vital organs such as the heart and brain, thereby increasing mortality risk [31]. Hyperglycemia also increases blood coagulability, heightening the likelihood of thrombotic events, especially during acute conditions like myocardial infarction and stroke, where blood clots can worsen outcomes [32,33]. Moreover, hyperglycemia triggers an inflammatory response, leading to increased levels of pro-inflammatory cytokines such as TNF-α, IL-6, and IL-1β. This inflammation can exacerbate existing health conditions and contribute to organ dysfunction and failure. Collectively, these factors can lead to arrhythmias and hemodynamic instability, ultimately increasing mortality risk in critically ill patients [28].

The SHR_24_ has not yet been proven to enhance the predictive capability of any models in the myocardial infarction and shock population. Our research further explores and enhances the predictive capabilities of the IABP-Shock II and CardShock scores, two of the most robust current risk models, for assessing in-hospital mortality risk among AMICS patients. Despite the robust performance of the IABP-Shock II and CardShock scores in predicting in-hospital mortality risk among patients with CS, these scoring systems have not been further studied specifically in AMICS patients. In a study by Miller et al. [34], within the AMICS subgroup, an observed trend toward enhanced discrimination was noted. Consistent with this, the validation study conducted by Rivas-Lasarte et al. [35] further affirmed the favorable predictive accuracy of both the IABP-Shock II and CardShock scores for assessing in-hospital mortality risk among AMICS patients. Our research echoes these findings, revealing that both scores possess relatively precise predictive abilities for in-hospital mortality risk among AMICS patients. Furthermore, we have incorporated the SHR_24_ and ΔBG_24_ into the aforementioned scoring systems, resulting in a significant improvement in the predictive accuracy of both the IABP-Shock II and CardShock scores. This modification is of paramount importance for refining existing scoring systems and improving their clinical utility, thereby facilitating timely clinical decision-making, such as determining the need for mechanical circulatory support device implantation in emergency catheterization laboratories.

## 5. Conclusions

In this study, the SHR_24_ presented good predictive value for short-term prognoses of the AMICS population, and a linear relationship was shown between the SHR_24_ and mortality. Furthermore, the SHR_24_ and ΔBG_24_ can significantly improve the accuracy of the IABP-Shock II and CardShock scoring systems, thereby assisting clinical professionals in assessing the prognosis of patients with AMICS.

### Strengths and Limitations

Firstly, although this is an observational and retrospective study, specialized physicians carefully reviewed every medical record of the cases to confirm that these selected patients met the diagnostic criteria strictly and ensured the data quality and completeness. Secondly, the data in our study were derived from a single center; the AMICS population included in this type of study is comparable to that in similar studies. Importantly, the present study novelly shows that SHR_24_ and ΔBG_24_ fluctuations significantly improve the current scoring systems for the AMICS population.

Of course, this study included a relatively small sample (*n* = 179) with an in-hospital mortality rate of approximately 60%. The limited sample size may reduce the statistical power and robustness of the prognostic analysis, while the extremely high mortality rate and some confounders (such as reperfusion timing) may introduce selection bias and limit the generalizability and external validity of our findings. This single-center retrospective study was conducted in a critically ill AMICS population with severe clinical conditions, which may further limit the extrapolation of our results to general AMI or low-risk patient cohorts. Therefore, larger multicenter prospective studies are warranted to further validate the clinical predictive value of the SHR24 and ΔBG_24_.

## Figures and Tables

**Table 1:** Baseline characteristics, medical history, and pre-existing conditions of all patients.

Characteristics *	Total (*N* = 179)	Non-Survivors (*N* = 104)	Survivors (*N* = 75)	T/Z/χ^2^	*p*-Value
Male (%)	118 (65.9)	67 (64.4)	51 (68.0)	0.248	0.618
Age (years)	65.47 ± 13.21	71.52 ± 12.48	64.29 ± 13.05	3.310	0.001
STEMI (%)	120 (67.0)	64 (61.5)	56 (74.7)	3.399	0.065
Previous MI (%)	44 (24.6)	27 (26.0)	17 (22.7)	0.255	0.613
Previous PCI (%)	46 (25.7)	31 (29.8)	15 (20.0)	2.195	0.138
Previous CABG (%)	5 (2.8)	3 (2.9)	2 (2.7)	0.139	0.709
Previous CVA (%)	26 (14.5)	16 (15.4)	10 (13.3)	0.148	0.701
HTN (%)	121 (67.6)	81 (77.9)	40 (53.3)	11.992	<0.001
T2DM (%)	58 (32.4)	38 (36.5)	20 (26.7)	1.939	0.164
CRF (%)	28 (15.6)	20 (19.2)	8 (10.7)	2.422	0.119
AF (%)	53 (29.6)	33 (31.7)	20 (26.7)	0.536	0.464
MR (%)	55 (30.7)	38 (36.5)	17 (22.7)	3.939	0.047
VSR (%)	9 (5.0)	8 (7.7)	1 (1.3)	2.478	0.115
VT/VF (%)	97 (54.2)	63 (60.6)	34 (45.3)	4.079	0.043
AVB (%)	22 (12.3)	12 (11.5)	10 (13.3)	0.130	0.718
IMV (%)	71 (39.6)	54 (51.9)	17 (22.7)	15.585	<0.001
IABP support (%)	81 (45.2)	43 (41.3)	38 (50.7)	1.528	0.216
LVEF (%)	35.26 ± 10.35	34.13 ± 11.11	36.82 ± 9.02	−1.697	0.092
SBP (mmHg)	70.15 ± 16.21	64.98 ± 14.74	77.44 ± 15.44	−5.414	<0.001
DSP (mmHg)	44.38 ± 13.78	39.88 ± 13.43	50.71 ± 11.68	−5.561	<0.001
HR (bpm)	108.64 ± 32.86	109.62 ± 35.78	107.26 ± 28.43	0.469	0.640
SI	1.67 ± 1.10	1.85 ± 1.37	1.42 ± 0.43	2.608	0.010
WBC (×10^9^/L)	18.49 ± 7.43	19.35 ± 7.66	17.29 ± 6.96	1.825	0.070
HCT	0.37 ± 0.07	0.35 ± 0.08	0.39 ± 0.06	−2.949	<0.001
Scr (μmol/L)	216.06 ± 135.89	273.4 ± 144.43	135.04 ± 62.93	8.640	<0.001
Alb (g/L)	34.75 ± 19.66	31.26 ± 11.50	39.88 ± 26.90	−2.846	0.005
ALT (IU/L)	109.5	488.00	72.00	−4.620	<0.001
Bilirubin (μmol/L)	16.20	16.36	16.20	−1.412	0.158
HbA1c (%)	7.05 ± 1.26	7.15 ± 1.15	6.90 ± 1.40	1.309	0.193

STEMI, ST-elevation myocardial infarction; PCI, Percutaneous Coronary Intervention; CABG, Coronary Artery Bypass Grafting; HTN, hypertension; CVA, cerebrovascular accident; T2DM, type 2 diabetes mellitus; CRF, chronic renal failure; AF, atrial fibrillation; MR, mitral regurgitation; VSR, ventricular septal rupture; AVB, atrial–ventricular block; IMV, invasive mechanical ventilation; IABP, intra-aortic balloon pump; LVEF, left ventricular ejection fraction; SBP, systolic blood pressure; DSP, diastolic blood pressure; HR, heart rate; SI, shock index; WBC, white blood count; HCT, hematocrit; Scr, serum creatinine; Alb, albumin; ALT, Alanine Aminotransferase; HbA1c, hemoglobin A1C; * Plus–minus values are means ± SD unless otherwise indicated.

**Table 2:** Characteristics of coronary anatomy of AMICS patients who received emergent coronary angiography.

	Total (*N* = 129)	Non-Survivors (*N* = 71)	Survivors (*N* = 58)	T/Z/χ^2^	*p*-Value
Multi-vessels (%)	60 (46.5)	31 (43.7)	29 (50.0)	0.515	0.473
LM (%)	29 (22.5)	14 (19.7)	15 (25.9)	0.691	0.406
LAD (%)	63 (48.9)	37 (52.1)	26 (44.8)	0.678	0.418
LCX (%)	9 (7.0)	8 (11.3)	1 (1.7)	4.480	0.034
RCA (%)	28 (21.7)	12 (16.9)	16 (27.6)	2.144	0.143
Stent implant (%)	44 (34.1)	18 (25.3)	36 (62.1)	5.387	0.020
TIMI 0–2 (%)	53 (41.1)	50 (70.4)	3 (5.2)	56.150	<0.001

LM, left main coronary artery; LAD, left anterior descending artery; LCX, left circumflex artery; RCA, right coronary artery; TIMI, thrombolysis in myocardial infarction.

**Table 3:** Multivariable Cox regression analysis of prognostic factors for AMICS.

	B	SE	Wald	HR (Exp(B))	95.0% CI for HR		*p*-Value
					Lower	Upper	
**VIS**	0.00	0.00	0.02	1.00	0.994	1.007	0.89
**SHR_24_**	0.81	0.22	14.31	2.25	1.479	3.432	<0.001
**CARDSHOCK**	0.35	0.10	11.49	1.41	1.157	1.727	<0.001
**HTN**	0.55	0.36	2.43	1.74	0.867	3.482	0.12
**T2DM**	−0.45	0.36	1.60	0.64	0.316	1.281	0.21
**VA**	−0.20	0.47	0.18	0.82	0.33	2.046	0.67
**IABP**	−0.11	0.32	0.13	0.89	0.477	1.668	0.72
**PCI**	−0.17	0.52	0.10	0.85	0.305	2.345	0.75
TIMI final	−0.43	0.13	11.70	0.65	0.508	0.832	<0.001
**SI**	0.153	0.238	0.412	1.165	0.731	1.857	0.521

VIS, vasoactive–inotropic score; SHR, stress hyperglycemia ratio; HTN, hypertension; VA, ventricular arrhythmia (including ventricular tachycardia and ventricular fibrillation); T2DM, type 2 diabetes mellitus; IABP, intra-aortic balloon pump; PCI, percutaneous coronary intervention; TIMI, thrombolysis in myocardial infarction; SI, shock index.

**Table 4:** ROC analysis of IABP-SHOCK II scoring, SHR_24_ + IABP-SHOCK II, ΔBG_24_ + IABP-SHOCK II; CardShock scoring, SHR_24_ + CardShock II and ΔBG_24_ + CardShock.

Scoring System	Sensitivity	Specificity	PPV	NPV	AUC	ΔAUC	Z	*p*
IABP-SHOCK II	0.913	0.413	0.68	0.775	0.700	--	--	--
SHR_24_ + IABP-SHOCK II	0.673	0.867	0.875	0.657	0.793	0.093	2.488	0.013
ΔBG_24_ + IABP-SHOCK II	0.587	0.867	0.859	0.602	0.780	0.080	2.408	0.016
CardShock	0.750	0.693	0.772	0.667	0.754	--	--	--
SHR_24_ + CardShock	0.721	0.880	0.893	0.695	0.846	0.091	3.031	0.002
ΔBG_24_ + CardShock	0.750	0.760	0.813	0.687	0.810	0.056	2.170	0.030

PPV, Positive Predictive Value; NPV, Negative Predictive Value.

## Data Availability

Datasets used or analyzed during the current study are available from the corresponding author on reasonable request.

## Abbreviations

AMI, acute myocardial infarction; AMICS, acute myocardial infarction; BG, blood glucose; SHR, stress hyperglycemia ratio; CS, cardiogenic shock; URL, upper reference limit; ROS, reactive oxygen species; LM, left main coronary artery; LAD, left anterior descending artery; LCX, left circumflex artery; RCA, right coronary artery; TIMI, thrombolysis in myocardial infarction; PPV, Positive Predictive Value; NPV, Negative Predictive Value; STEMI, ST-elevation myocardial infarction; PCI, Percutaneous Coronary Intervention; CABG, Coronary Artery Bypass Grafting; HTN, hypertension; CVA, cerebrovascular accident; T2DM, type 2 diabetes mellitus; CRF, chronic renal failure; AF, atrial fibrillation; MR, mitral regurgitation; VSR, ventricular septal rupture; AVB, atrial–ventricular block; IMV, invasive mechanical ventilation; IABP, intra-aortic balloon pump; LVEF, left ventricular ejection fraction; SBP, systolic blood pressure; DSP, diastolic blood pressure; HR, heart rate; SI, shock index; WBC, white blood count; HCT, hematocrit; Scr, serum creatinine; Alb, albumin; ALT, Alanine Aminotransferase; HbA1c, hemoglobin A1C.
